# Preliminary study on heart response and locomotor parameters in Donkeys (*Equus asinus*) during exercise using fitness tracker (Equimetre)

**DOI:** 10.1038/s41598-024-72605-7

**Published:** 2024-09-27

**Authors:** Taleb Al Khamis, Turke Shawaf, Wael El-Deeb, Adel Almubarak, Mohammed Ali Al-Ali, Meshari Almuaqqil, Ahmad AlAiyan, Abdelgadir M. Homeida

**Affiliations:** 1 Institute for Research and Medical Consultations (IRMC), Imam Abdulrahman Bin Faisal University, 31441 Dammam, Saudi Arabia; 2 Department of Clinical Sciences, College of Veterinary Medicine, King Faisal University, 31982 AL-Ahsa, Saudi Arabia; 3 Department of Internal Medicine and Infectious Diseases, Faculty of Veterinary Medicine, Mansoura University, Mansoura, Egypt; 4 Shelter Units, Administration of Terrestrial Wildlife Conservation, National Centre for Wildlife, 12411 Riyadh, Saudi Arabia; 5 Department of Veterinary Medicine, College of Food and Agriculture, Emirates University, Al Ain, UAE

**Keywords:** Donkey, Electrocardiograms, Equimetre, Standard, Troponin, Tracker, Physiology, Zoology, Cardiology

## Abstract

The welfare of donkeys remains a compelling subject for researchers, with limited literature available on the response of the donkey cardiovascular system during strenuous exercise. The study aimed to address two primary objectives. Firstly, to assess the reliability of wearable devices in detecting heart rate (HR) and ECG readings. Secondly, to determine HR, locomotor and cardiac troponin 1 (cTnI) levels in donkeys during exercise. A total of seven donkeys were outfitted with two systems for ECG measurements, namely Equimetre and the Standard base apex, to enable a comparison between the two. Additionally, fifteen apparently healthy donkeys equipped with Equimetre were divided into two groups: the race group (R), consisting of donkeys trained for racing, and the non-race group (NR), comprising donkeys used for regular riding. The results indicated a level of agreement between the two devices in intervals R-R (P =  < 0.0001), S-T (P = 0.0002), Q-T(P = 0.0003), P-R (P = 0.0037), segment P-R (P = 0.0023) and HR (P =  < 0.0001) at rest. This suggested that Equimetre can provide a level of accepted ECG reading in donkey. No significant difference in heart response and locomotor parameters between donkey groups, although this finding needs further studies to verify it and to understand the dynamics of donkey. This study demonstrates the feasibility of Equimetre in detection HR and present initial data of heart response and locomotor in donkeys during exercise.

## Introduction

Donkeys have played a significant role in transportation in the past, and they continue to serve as vital pack animals today. Additionally, they hold cultural significance for certain societies^1^. Welfare of donkey still an interesting topic for researcher in developing countries^2^ .Normal electrocardiographic parameters in donkeys were studied previously at rest^3^ and during long term record^4^. Moreover, cardiovascular diseases and cardiac rhythms in donkey were previously studied^4,5^. However, there are few studies on performance of donkey under exercise^6–8^ and none of these studies have focused on cardiovascular and locomotor data^9^.

In the veterinary field, there has been a growing trend in integrating wireless electrocardiogram (ECG) monitoring to assess cardiovascular parameters, and studies have emerged to validate their reliability in various species^10^. Portable ECGs have been recently investigated in dogs^11,12^ , ruminants^13,14^ , camels^15^, and horses^16,17^ . Nevertheless, there is still a need for validating the data from wearable devices and evaluating the accuracy and reliability of these devices' measurements^10^. Equimetre is new technology developed as horse fitness tracker and been previously used for heart rate detection and performance parameters collection in horse^18^. Moreover, Equimetre was validated for ECG and arrhythmia recognition in horse^16^.

There is limited information available in the literature regarding the response of donkey cardiovascular system during strenuous exercise. In the Kingdom of Saudi Arabia, sports animal sector is expanding, and there is a growing interest among owners in various sporting disciplines for different animal species. As part of this trend, local donkey race competitions in Saudi Arabia, which involve free riding and cart racing similar to harness or horse driving, are organized by owners annually^19^. These races typically cover distances ranging from 400 to 1000 m. The objective of this study is twofold. Firstly, to evaluate the reliability of the Equimetre fitness tracker in detecting ECG and heart rate (HR) in apparently healthy donkeys at rest. Secondly, to determine the difference in heart rate (HR) before and after exercise as measured by the Equimetre fitness tracker and heart enzymatic activity-cardiac troponin 1 (cTnI) levels in both race-trained and untrained donkeys.

## Materials and methods

### Animals

A total of seventeen apparently healthy donkeys from private farms in Al-Hasa Eastern Saudi Arabia, age ranging from 20 months to 19 years old were included in this study, consisting of twelve males and five females. The morphometric characteristics of these local Saudi Arabia donkeys was previously described by^1^. Study was conducted in Al-Hasa, Eastern Saudi Arabia. Prior to enrollment, the donkeys underwent clinical examination, and their medical history was assessed to confirm their overall health. Throughout the study, the donkeys were kept in stall barns and provided with free access to feed and water under the care of their respective owners.

### Equipment

The study was conducted in two phases.

### Comparison between two ECG systems

**Fig. 1:**
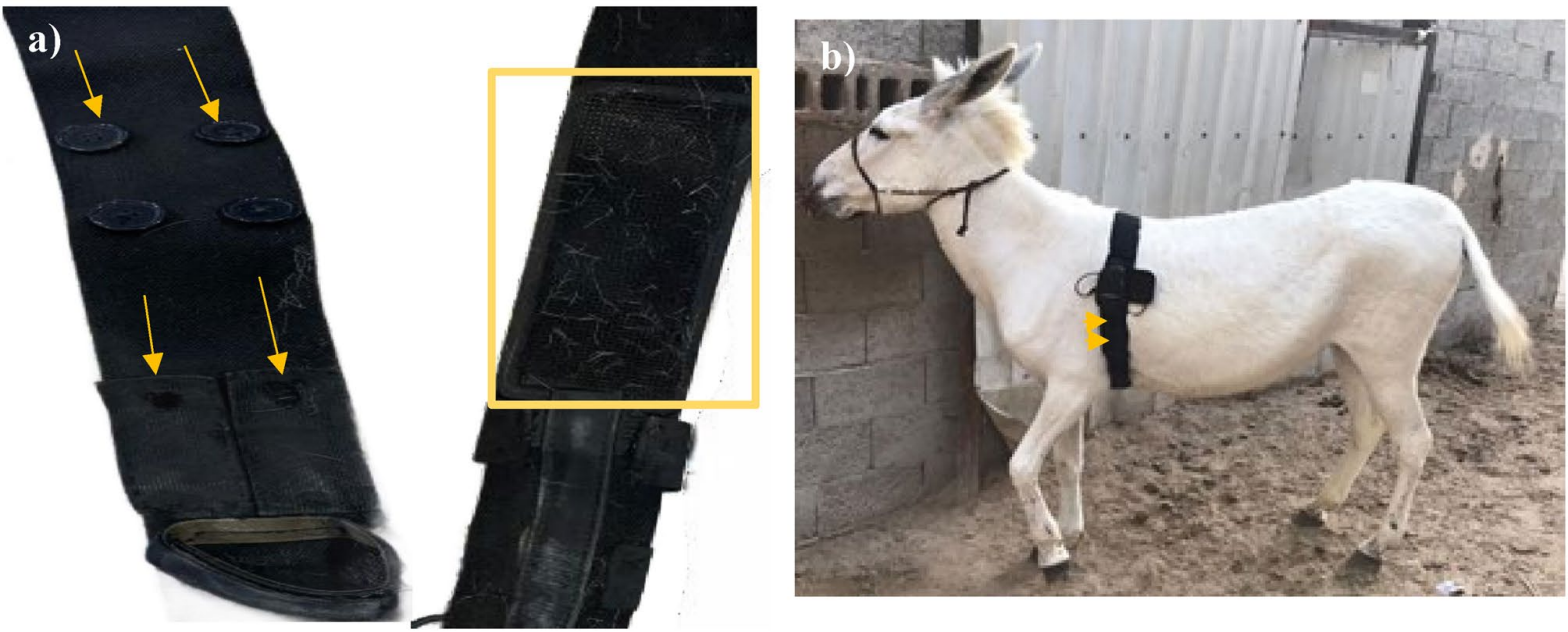
(**a**) shows modification on girth, arrows show button and buttonhole outside and square shows electrode from inside (**b**) Shows fixed Equimetre girth on female donkey, arrow shows secure area.

A total of seven male donkeys were included in this study, and they were equipped with two systems for comparison: the Equimetre (Arinoneo, Paris, France) and the standard base-apex ECG (BM7 VET; Bionet, Republic of Korea). The Equimetre system consisted of a two-electrode sensor attached to an elastic girth, while the standard base-apex ECG utilized surface electrodes attached to the skin using alligator clips. All donkeys were positioned in a standing position and were not sedated during the measurements. The first electrode of the Equimetre girth was positioned on the left side of the chest, just behind the elbow joint, at the cardiac apex. The second electrode was positioned behind the scapula on the thoracic wither region. The girth was modified as necessary to fit the donkey's chest circumference and securely hold the electrodes in place (Fig. 1).

**Fig. 2:**
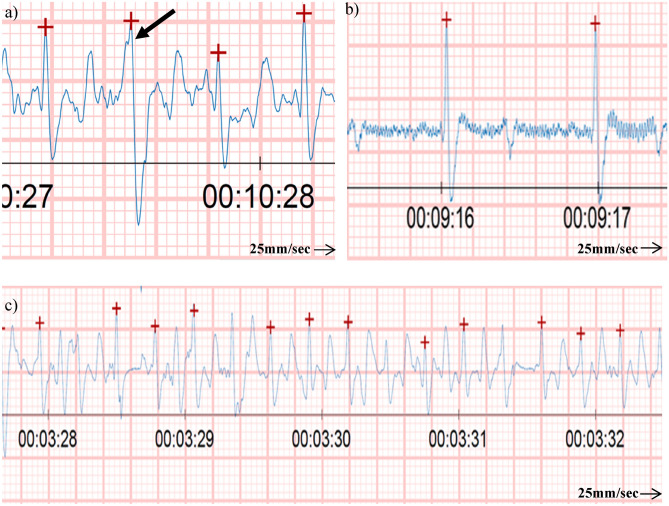
Shows noise in ECG trace from Equimetre (**a**) Movement artifact at rest (**b**) Electromagnetic interference at rest (C) Shows normal complexes and motion artifacts during exercise.

For comparison purposes, all standard base-apex electrodes were positioned on the left side of the donkey. The left arm electrode (positive) was placed above the olecranon, in close proximity to the first Equimetre electrode^20^. The right arm electrode (negative) placement was modified and positioned two-thirds of the way down the mane of the neck on the left side, aligned parallel to the Equimetre electrodes. The third electrode was placed cranially and parallel to the second thoracic girth electrode of the Equimetre on the left side of the chest in wither area^20^. The Equimetre electrodes were moistened with tap water in accordance with the manufacturer's instructions to maintain electrical conductivity. Additionally, gel was applied to the electrodes of the standard base-apex system (Fig. 2).

### Performance assessment

Fifteen apparently healthy donkeys, all equipped with Equimetre, were divided into two groups. The race group (R) these donkeys underwent different training methods by owners in terms of training and resting day or days/ week, distance range from 500 to 2000m, work intensity from trot to canter, free riding or small cart on dirt or sand surfaces. The R group consisted of eight male donkeys (N = 8) that were specifically trained for racing and had a previous history of participating in races. Two donkeys from the R group experienced a failure in collecting heart and locomotor parameters due to the loss of girth contact with skin. Non-race group (NR) comprised 5 female (N = 5) and 2 males (N = 2), all used for regular riding once a week at least. Exercise was taking place in different day for R group and NR group during routine training and riding of each donkey group for criteria of distances ranged 400m minimum –1000m maximum and work intensity reached to canter phase. Exercise parameters that include heart rate at rest (Before), heart rate after exercise cessation (After) and heart rate maximum during exercise were collected by Equimetre. Moreover, Equimetre record locomotor parameters (speed maximum (Km/h), max stride frequency strids per second (stride/s), max stride length meter per stride (m/stride) and total distance during exercise. Blood samples immediately before exercise and shortly after exercise cessation were collect and serum samples were stored at -80 ℃ until analyzed for cTnI levels.

### Data collection & analysis

The Equimetre device linked to Equimetre ECG application on smartphone iPhone 7 (Apple, USA). The ECG was displayed live digitally at paper speed 25 mm/s application and ECG tracing was recorded automatically. For the standard base-apex tracing ECG was viewed and printed at paper speed of 25mm/s with gain of 20 mm/mV using lead Ш. Two to three good ECG reading of 15s traces was obtained from each donkey for each device and tracing time setup was recorded for both devices at the same time. Raw data of ECG exported files from Equimetre was downloaded as previously described^15^ for complexes view & subtraction of non-recorded ECG time. All tracings were reviewed for baseline artifacts of P wave, QRS complex and T wave segments^17^. All measurements were recorded by one operator.

For the purpose of system comparison, a 15-s trace of the reviewed ECG tracing in total of 20 traces was selected for analysis. Each tracing was anonymized and randomized before being evaluated for the measurement of normal ECG variables. These variables included heart rate (HR) in beats per minute, which was calculated by multiplying the number of PQRST complexes from the 15-s trace by 4. Other measures included the P-R interval (in seconds), QRS duration (in seconds), R-R interval (in seconds), Q-T interval (in seconds), S-T interval, P-R segment, and S-T segment (all in seconds). Additionally, the amplitudes for the P-Peak, Q-Peak, R-Peak, S-Peak, and T-Peak were measured in millivolts. For each measurement, the first and last three complexes from the 15-s ECG trace were taken into account, and the average of these measurements over the three complexes was used for analysis. Measurements done by hand for both Equimetre and standard ECG systems^21^.

The exercise data, including heart rate (before exercise and after exercise) and maximum heart rate during exercise, as well as locomotor parameters such as maximum speed (in km/h), stride frequency (in strides per second), and stride length (in meters per stride), were extracted from the Equimetre platform in Excel format for statistical analysis^18^.

### Cardiac troponin I

Cardiac troponin analysis was conducted on samples from 14 donkeys as per manufacture instructions, including both the **R** and **NR** groups. However, one sample from the R group was excluded due to hemolysis. The analysis was performed using a two-site sandwich ELISA method with the Horse Cardiac Troponin I ELISA Kit (MBS017380, My BioSource, USA). The kit has a detection range of 0.25 ng/mL to 8 ng/mL and a sensitivity of 0.1 ng/mL^22^.

### Statistics analysis

Donkeys’ characteristics at along with heart response and locomotors parameters measured before exercise were summarized using descriptive statistics and stratified non-race and race group. Median and range were used to describe continuous variables whilst frequency and proportion described categorical variables. Potential differences between race and non-race donkeys were tested using Wilcoxon rank test for continuous variables and Chi-squared test for categorical variable. The performance between Standard base-apex and Equimetre was evaluated by comparing the summary measure within healthy donkeys. Intraclass correlation coefficient (ICC) was used to assess the agreement between the two methods of ECG trace. For each donkey, the average measurements over the three examiners for Standard and Equimetre respectively. The ICC was then calculated based on the average measurement from each donkey. ICC values were interpreted for values ≤ 0.20, 0.21–0.40, 0.41–0.60, 0.61–0.80, 0.81–0.99, and 1 as having a weak correlation, fair, moderate, good, very good, and perfect agreement, respectively, as described previously^13^ .All statistical analyses were performed using R version 3·6·1 (R Core Team, Vienna, Austria). Two-sided p-values < 0.05 were considered statistically significant.

## Result

### Feasibility of ECGs trace

**Fig. 3:**
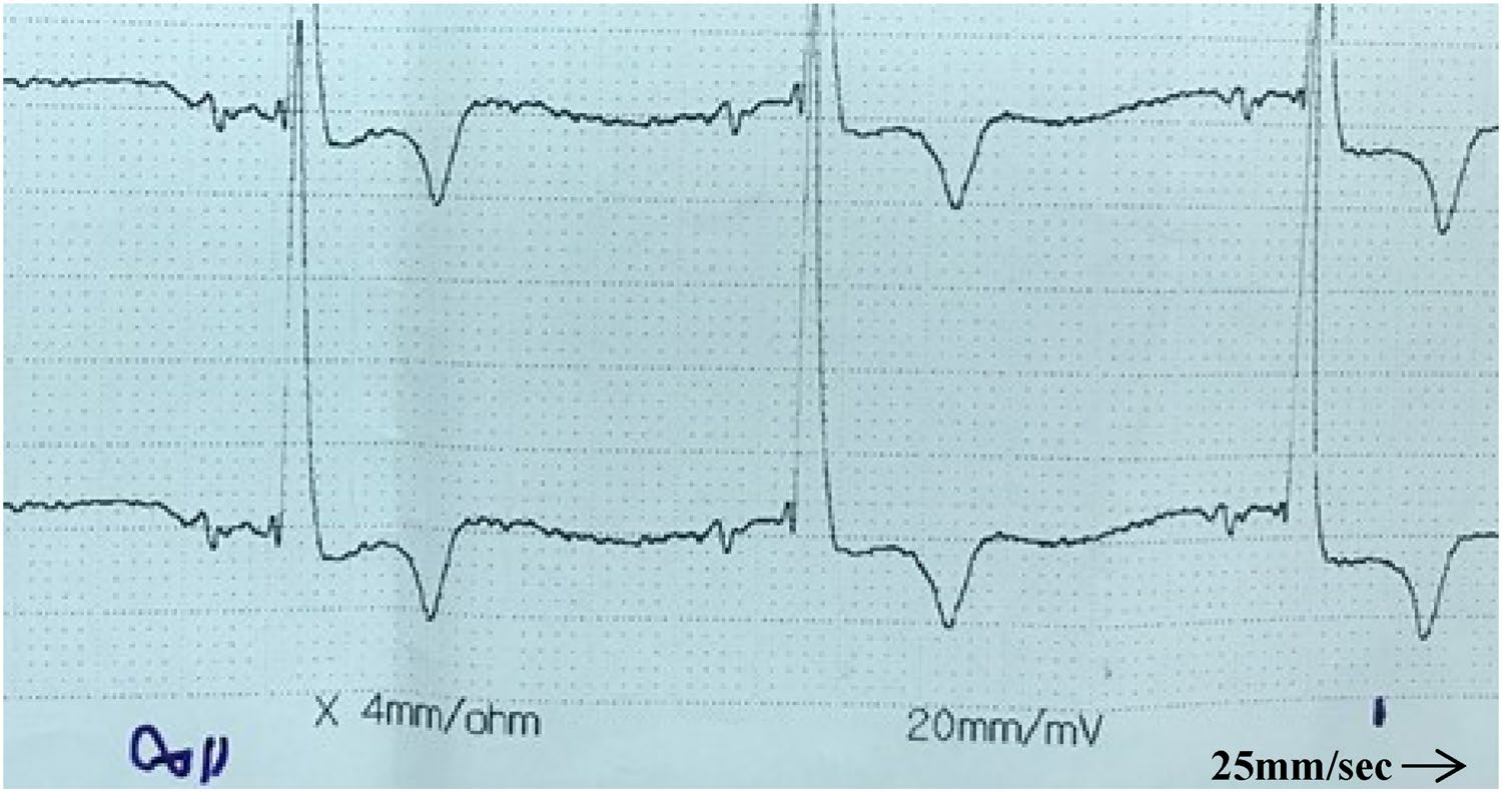
shows standard base-apex ECG trace.

Feedback from operator on fixing time of the Equimetre girth electrodes on donkeys was observed to be shorter compared to the standard base-apex system alligator electrodes grip side frequently loss while animal moving. Consequently, at rest Equimetre displayed consistent stability in ECG traces with fewer movement artifacts compared to the standard base-apex system. ECG trace evaluator noticed difference in the polarity of ECG complex variables between the two systems. Equimetre system showed either complete positivity or biphasic morphology T waves, in contrast most traces from standard base-apex system showed negative T waves and few traces of positive waves. Moreover, ECG trace from Equimetre showed bifid P waves and less prominent P peaks. Complex morphology from Equimetre traces displayed waves P-QRS-T, in contrast to standard base-apex traces showed absent of S wave Fig. 3. Overall, Complex from Equimetre traces showed short peaks amplitude in compared to the standard base-apex system.

Traces from Equimetre ECG during exercise showed different types of noise that includes movement artifacts in all donkey records Fig. 2a.; however, clear and normal morphological P-QRS-T complexes can be detected Fig. 3c. Additionally, two cases of electromagnetic interference and low voltage signals in the ECG were observed during exercise traces in all donkey records Fig. 3b.

### Heart rate (HR) and complex measurements

Table.1 shows the median and range of heart rate (HR) in healthy donkeys measured for both devices. The standard base-apex method yielded a HR measurement of 47 (range: 24–75), which was very close to the Equimetre group with a median HR of 48 (range: 24–72). The agreement between the two methods standard base-apex and Equimetre measurements shows in Table.2 for heart rate average. In apparently healthy donkeys, there was a very good HR measurements correlation (ICC = 0.99, P < 0.0001*), with a 95% confidence interval ranging from 0.99 to 1.

**Table 1:** Summary of ECG Measurements for Healthy Donkeys.

Measurement	Standard base-apex median (range)	Equimetre vet median (range)
Heart rate(bpm)	47 (24, 75)	48 (24, 72)
P-R interval (s)	0.21 (0.15, 0.29)	0.21 (0.12, 0.28)
QRS duration (s)	0.12 (0.11, 0.15)	0.13 (0.12, 0.14)
S-T interval (s)	0.34 (0.29, 0.42)	0.35 (0.30, 0.45)
Q-T interval (s)	0.47 (0.41, 0.61)	0.48 (0.42, 0.58)
P-R segment (s)	0.13 (0.09, 0.21)	0.13 (0.08, 0.21)
S-T segment (s)	0.21 (0.15, 0.29)	0.25 (0.20, 0.33)
R-R interval (s)	1.29 (0.81, 2.59)	1.27 (0.79, 2.33)
P-Peak (mv)	0.04 (− 0.02, 0.10)	0.03 (-0.02, 0.07)
Q-Peak (mv)	0.00 (− 0.08, 0.08)	− 0.06 (− 0.11, − 0.03)
R-Peak (mv)	2.07 (1.12, 3.18)	1.07 (0.87, 1.36)
S-Peak (mv)	0.10 (0.05, 0.15)	− 0.29 (− 0.44, − 0.13)
T-Peak + (mv)	0.04 (− 0.05, 0.38)	0.24 (0.11, 0.30)
T-Peak- (mv)	0.00 (0.00, 0.40)	− 0.08 (− 0.14, 0.00)

*s* second, *mv* Millivolt, *bpm* Beat per minute.

**Table 2:** Intraclass Correlation Coefficient (ICC) Agreement between Two Methods of ECG Trace.

Variable (Average)	ICC	P-value	95% CI
Healthy donkeys ECG trace			
Heart rate (bpm)	0.99	< 0.0001*	(0.99, 1)
Average PR (s)	0.58	0.0037*	(0.19, 0.82)
QRS duration (s)	− 0.15	0.7331	(− 0.56, 0.32)
S-T interval (s)	0.71	0.0002*	(0.39, 0.88)
Q-T interval (s)	0.70	0.0003*	(0.37, 0.88)
P-R segment (s)	0.61	0.0023*	(0.22, 0.83)
S-T segment (s)	0.16	0.2528	(− 0.31, 0.57)
R-R interval (s)	0.97	< 0.0001*	(0.92, 0.99)
P-Peak (mv)	0.00	0.4982	(− 0.45, 0.45)
Q-Peak (mv)	− 0.39	0.9520	(− 0.71, 0.08)
R-Peak (mv)	− 0.47	0.9801	(− 0.76, − 0.02)
S-Peak (mv)	− 0.94	0.9997	(− 0.98, − 0.86)
T-Peak + (mv)	0.05	0.4173	(− 0.41, 0.49)
T-Peak- (mv)	− 0.89	0.9026	(− 3.95, 0.29)

*s* second, *mv* Millivolt, *bpm* Beat per minute.

P- value was set for (p < 0.05) of significance*.

Table 1 displays the median and range of P-R, QRS, S-T, Q-T, and R-R intervals for each donkey, measured using both the standard base-apex system and the Equimetre. The agreement between the two methods standard base-apex and Equimetre measurements shows in Table.2 for complex intervals.

For apparently healthy animals, a moderate correlation was found for the average P-R interval (ICC = 0.58, P = 0.0037, 95% confidence interval: -0.19 to 0.82). Good correlations were observed for the average S-T and Q-T intervals (ICC = 0.71, P = 0.0002) and (ICC = 0.70, P = 0.0003) respectively. The average P-R interval showed a moderate correlation (ICC = 0.58, P = 0.0037). Notably, a very good correlation was observed in the R-R interval (ICC = 0.97, P = 0.0001).

Table.1 presents the median and range values of the P-R and S-T segments for donkeys, measured using both the standard base-apex and Equimetre devices. The agreement between the two methods standard base-apex and Equimetre measurements shows in Table.2. A good correlation was observed for the P-R segment (ICC = 0.61, P = 0.0023), indicating an agreement between the two methods. However, the average correlation for the S-T segment was weak (ICC = 0.16, P = 0.2528), suggesting a lower level of agreement between the devices for this segment.

Table 1 presents the median and range values of P, Q, R, S, and T peaks for all donkeys, measured using both the standard base-apex system and the Equimetre. Table.2 demonstrates the agreement between the two methods of complex peaks between the two devices. The average correlation between the two methods for ECG trace measurements of P, Q, R, S, and T peaks was weak agreement between the two devices.

**Fig. 4:**
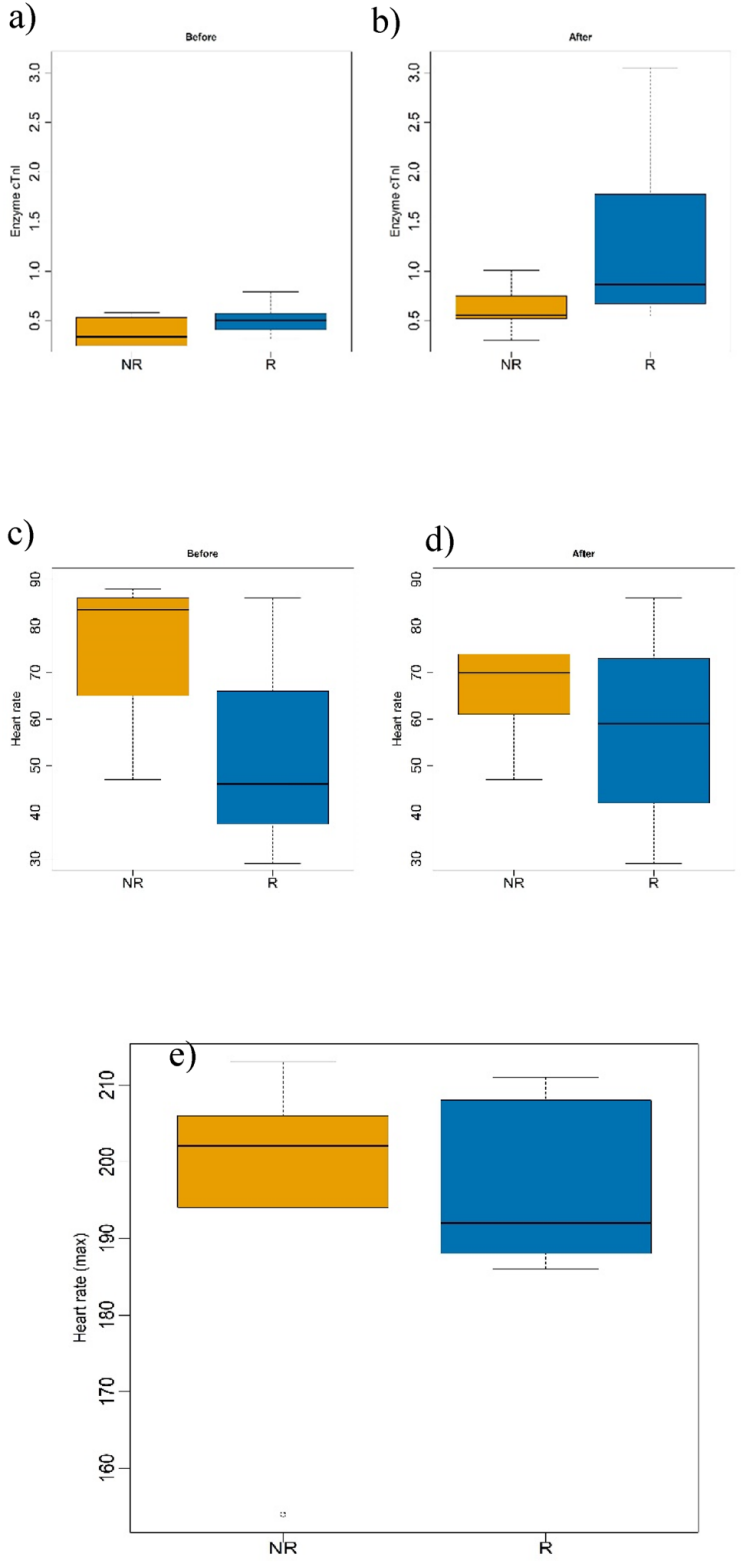
Boxplots Comparing Heart Parameters between Race Donkeys (R) and Non-Race Donkeys (NR) (**a** & **b**) Cardiac Enzyme cTnI Activity Before and After Exercise (**c** & **d**) Heart Rate Range at Rest (Before) and After Exercise (**e**) Maximum Heart Rate Range During Exercise Statistical values are presented in Table 3.

There were no statistically significant differences observed between the non-race and race group of donkeys in heart response and locomotors parameters, as shown in Table 3. Difference in gender variable attributed to the number of males to female's ratio in the sample. Figure 4. showed differences in heart rate (HR) variables (c&d) and cardiac troponin I levels (a&b) between race-trained and untrained donkeys. No statistical difference in heart response during exercise between the groups, however numerically NR group showed high medians heart rate parameters (Before = 83.5 bpm, after = 70.0 bpm and Max = 202.0 bpm) compared to R group (Before = 46.0 bpm, after = 59.0 bpm and Max = 192.0 bpm). One case of odd heart rate max of 273 bpm was removed from the analysis. Activity of cardiac troponin I was low in NR donkey (median = 0.3 ng/ mL before—0.6 ng/ mL after) compared to R group (median = 0.5 ng/mL before—0.9 ng/mL after).

**Table 3:** Comparative Analysis of Donkey Characteristics, Locomotor Parameters, Heart Parameters, and Cardiac Activity: Stratification by Race and Non-Race.

Variable	Non-racing donkey median (range)	Race donkey median (range)	P-value
Gender			0.0034*
Female n (%)	5 (71%)	0 (0%)	
Male n (%)	2 (29%)	8 (100%)	
Age (years)	8 (2, 18)	3 (2, 19)	0.4128
Total exercise distance(m)	620 (415, 870)	673 (415, 875)	0.7339
Max Speed (km/h)	26.7 (11.1, 31.5)	26.4 (20.1, 36.3)	0.5654
Max stride length (m/str)	3.2 (1.6, 3.6)	3.0 (2.5, 4.1)	0.5462
Max Stride Frequency (stride/s)	2.5 (1.9, 2.8)	2.5 (2.2, 2.6)	0.9714
Heart rate (Before)	83.5 (47.0, 88.0)	46.0 (29.0, 86.0)	0.1745
Heart rate (After)	70.0 (47.0, 108.0)	59.0 (29.0, 86.0)	0.2746
Heart rate (Max)	202.0 (154.0, 213.0)	192.0 (186.0, 211.0)	0.8675
Enzyme cTnl (Before)	0.3 (0.3, 0.6)	0.5 (0.3, 0.8)	0.1533
Enzyme cTnl (After)	0.6 (0.3, 1.0)	0.9 (0.5, 3.1)	0.0728

cTnI level is in ng/mL.

*m* Meter, *Max* Maximum, *Km/h* Kilometre per hours, *stride/s* Strids per second, *m/stride* Meter per stride.

P- value was set for (p < 0.05) of significance*.

**Fig. 5:**
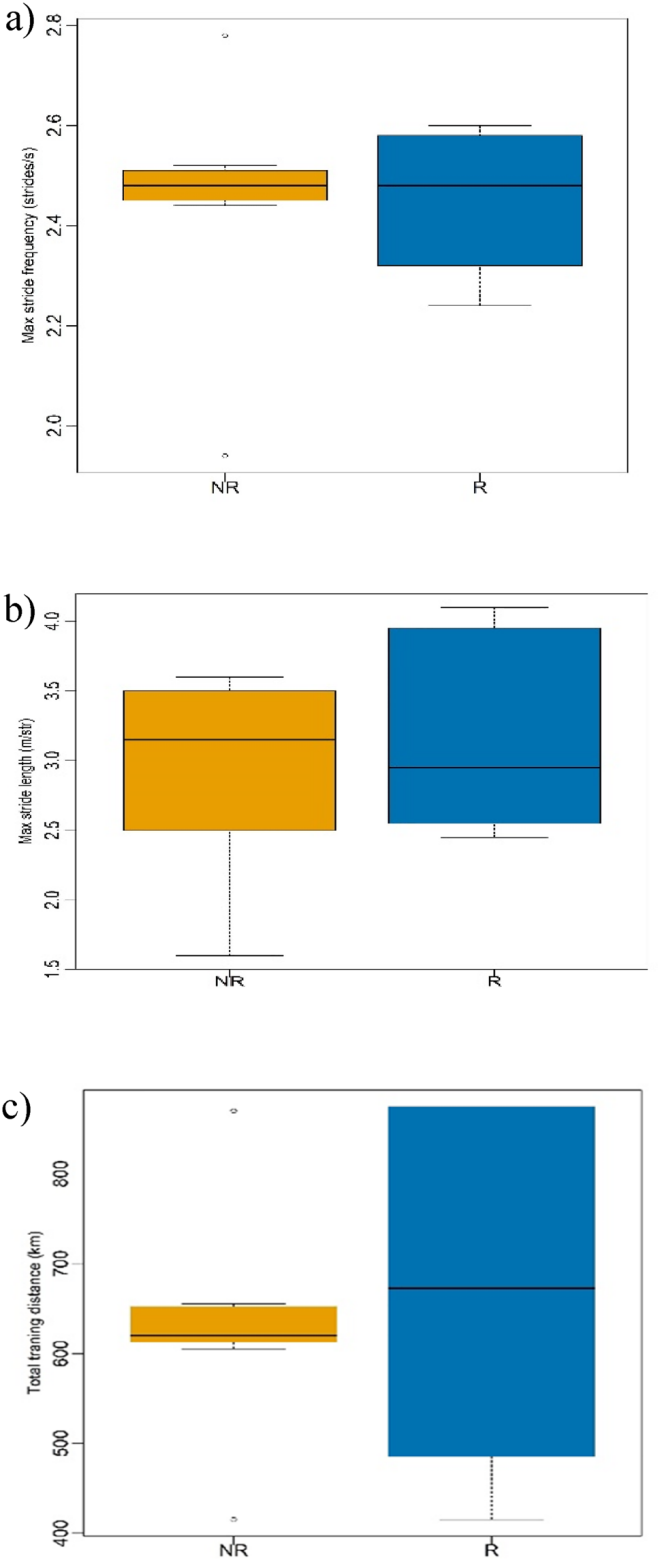
Boxplots Comparing Locomotor Parameters between Race Donkeys (R) and Non-Race Donkeys (NR) during Exercise (**a**) Maximum Stride Frequency (strides/second) Range (**b**) Maximum Stride Length (meters/stride) Range(c) Maximum Speed (kilometers/hour) Range Statistical values are presented in Table 3.

Figure 5 presents the descriptive data for locomotor parameters. The race and non- race groups showed a less difference in median of stride length (non-race = 3.2 m/stride; Race = 3 m/stride).

## Discussion

To the authors best knowledge this is the first report of using fitness tracker Equimetre for recording heart electrical activity at rest and under exercise in donkeys. Wearable devices are profound as valuable tools in health monitoring for observation, diagnosis and treatment, ultimately improving life quality^23^. Commercial activity trackers showed a benefit at both clinical and non-clinical levels. However, their continuous evaluation and research are necessary due to the need for constant redesign and upgrades^24,25^. The present study demonstrated the feasibility of Equimetre to obtain ECG and heart rate (HR) in donkey. Result shows significant agreement between two systems in heart rate and ECG variables R-R, S-T, Q-T, P-R intervals and P-R segments at rest. This suggests that Equimetre could provide a sufficient quality of ECG trace for detection heart rate and trace evaluation for diagnosis in donkeys. Additionally, the study present data of heart response during exercise electrocardiography and enzymatic activity in trained donkey and other uses for regular ride. Furthermore, this study report locomotor parameters during exercise for the first time as far to authors knowledge.

In this study, fitness tracker Equimetre observed to have a better stability in obtaining ECG traces from resting donkeys. This stability can be due to girth design of Equimetre system which electrodes securely wraps around the donkey chest. This observation aligns with pervious study that found band increased pressure in electrodes and this improving electrodes contact with skin^26^. Quality of ECG during exercise was found low and showed noise artifacts, electromagnetic interferences, and low signals. This observation can be explained by the difficulty in maintaining contact of electrodes to skin in the absence of saddle this may attributed to the characteristics of girth design to be under saddle in horse riding and/or electrodes size and distance design to fit horse chest. This finding supported by suggestions from previous studies that major artifacts in ECG recordings could be associated with poor electrode contact to skin and various types of movement^27,28^. This finding needs to be investigated further for species-to-species variations in girth design and characteristic.

Several studies reported variation between donkey breeds in ECG normal values and standardized electrocardiographic parameters for comparison are needed^4,29,30^.To our best knowledge, this study is describing ECG measurements for first time in local Saudi Arabia donkey. Therefore, the ECG parameters are compared with the available data in literatures for other breeds. This study demonstrated results of ECG measurements in donkeys for heart rate, ECG waves and polarity. Polarity of T waves in this study showed inconsistency between the two ECG systems. A negative T waves observed in our study in most standard base-apex trace are in line with the previous studies by Rezakhani and Yazdanmehr^3^ and Escudero^30^ and present of positive T waves supported previous report by^4^ that presence of an inversions T shape polarity in donkey during 24-h Holter record . Present of positive and biphasic T waves in Equimetre traces consistent with previous study in Pega breed donkey^29^ . The different in the polarity between ECG devices is in agreement with what previously reported in studies comparing standard ECG devices with new portable ones^21^ and evaluation of smartphone based ECG reader^27^.

Bifid P waves result in this study is consistent with results previously reported^29,30^ and the amplitude was more prominent in Equimetre compared to standard base apex systems. This can be explained by the placement of standard base apex negative electrode on the left side and parallel to Equimetre electrodes. Consequently, results from standard base-apex system P wave amplitude incomparable. Our findings from in donkey showed different electrocardiographic parameters than what reported previously in literatures for other donkey breeds^29,30^. Generally, our finding on complex waves amplitudes from Equimetre compared to standard bas apex were shorter. This may be attributed to the differences in electrodes material and placement. This finding may suggest further investigation on electrode placement and material are warrant in donkey as previous studies reported that electrode placement, electrode material, and skin–electrode contact impedance can affect wave amplitudes^26,27^. Duration of the QRS wave was relatively similar between the devices numerically and did not reach statistical significance. This finding on local donkey QRS duration were not in agreement with previous study on Zamorano-leones breed^30^ but convenient to what been reported on Pega breed donkey^29^ . This could be explained by the difference in ECG trace record setting used in this study and the variations in age, gender, and breed examine donkey. Influence of breed, sex and breed on electrocardiographic parameters are previously mentioned^29,30^ and large studies on those factors from different donkey breeds to provide an inside information were suggested.

Despite Equimetre results showed minimum correlation in peaks amplitude and segments, the significant agreement between two system in intervals suggested that Equimetre can provide a level of accepted ECG reading in donkey. Additionally, significant agreement in resting heart rate between the two devices suggests that the Equimetre is accurate and reliable as it align with previously reported ranges in other studies^30,31^.

To the best of authors knowledge, no previous study has compared different types of donkey activity and their relationship to physiological responses of heart. Therefore, presented data in this study is reported for the first time and further studies are needed for validation and comparison. In this study R group exhibited lower median heart rates compared to NR group donkeys. This suggest that work type and load may have influence on donkeys heart adaption. This observation is consistent with pervious study in horse comparing trained and untrained horses^32^. Studies on donkeys form different work types, age and sex are needed to provide better information on response of heart autonomic nervous system.

Cardiac enzyme troponin I values from NR group were lower than those of R group donkeys numerically. Previous studies in racehorses found that exercise and racing increased cTnI level after workload^33,34^ this may explain our finding in the difference between groups. Locomotor results from R group showed no significant variability between parameters compared to the NR group, although this finding need further studies to verify it and to understand the dynamics of donkey. This study has some limitation that may influence presented data includes age range of animal, gender and the variation of training level and method of animal.

In conclusion, this study demonstrates the feasibility of Equimetre in capturing electrocardiogram (ECG) and collecting performance data in donkeys. The preliminary findings of exercise in this study provide initial data for heart response and locomotor in donkey. Further studies are needed to optimization Equimetre to fit species characteristics and understand donkey heart response and locomotor dynamics.

## Data Availability

The datasets for this study can be made available by the corresponding author without undue reservation.
